# Supplementary material for: Recyclable Composite Membrane of Polydopamine and Graphene Oxide-Modified Polyacrylonitrile for Organic Dye Molecule and Heavy Metal Ion Removal
Source: Membranes (Basel). 2022 Sep 27;12(10):938. doi: 10.3390/membranes12100938 (PMC9609451; doi:10.3390/membranes12100938)
Supplement: Supplementary file 1 [file membranes-12-00938-s001.zip › membranes-1870322-supplementary.pdf]

membranes

Supplementary Material

Recyclable composite membrane of polydopamine and graphene oxide modified polyacrylonitrile for organic dye molecules and heavy metal ion removal

Haoyu Wang [1], Zhiyun Han [1], Yanjuan Liu [1], Maojin Zheng [1], Zhenbang Liu [1,*], Wei Wang [1], Yingying Fan [1,*], Dongxue Han [1 2], and Li Niu [1]

[1] Center for Advanced Analytical Science, Guangzhou Key Laboratory of Sensing Materials & Devices, School of Chemistry and Chemical Engineering, Analytical and Testing Center, School of Computer Science and Cyber Engineering, Guangzhou University, Guangzhou 510006, PR China
[2] Guangdong Provincial Key Laboratory of Psychoactive Substances Monitoring and Safety, Anti-Drug Technology Center of Guangdong Province, Guangzhou 510230, PR China
* Correspondence: cczbliu@gzhu.edu.cn (Z.L.); ccyyfan@gzhu.edu.cn (Y.F.)

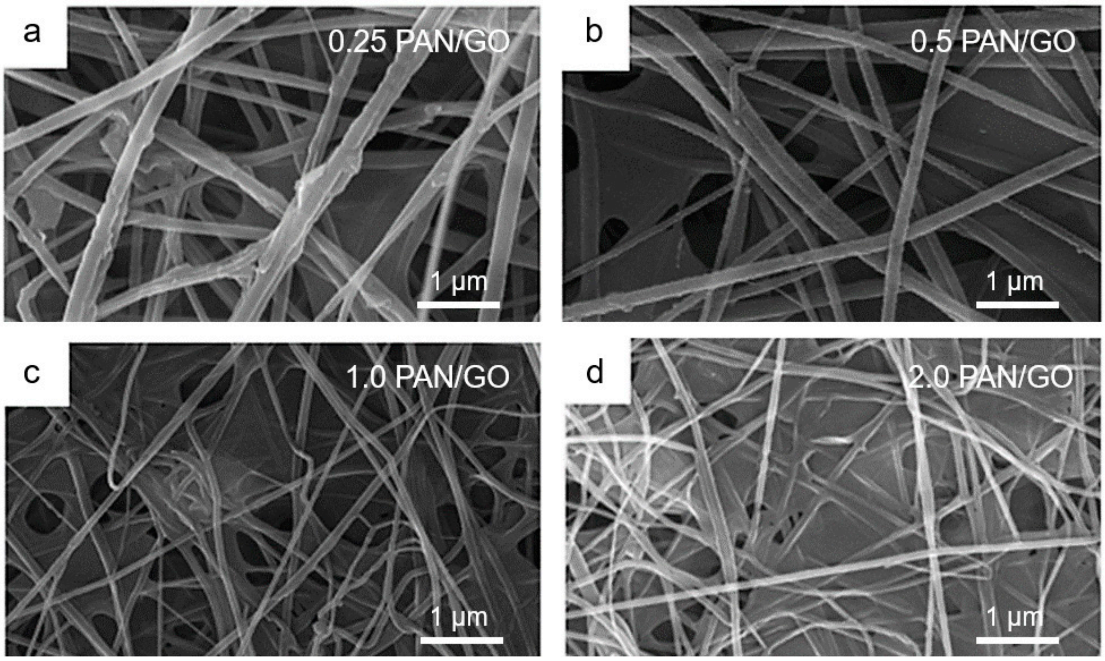

Figure S1. SEM images of （**a**） 0.25 PAN/GO, （**b**） 0.5 PAN/GO, （**c**） 1.0 PAN/GO and （**d**） 2.0 PAN/GO.

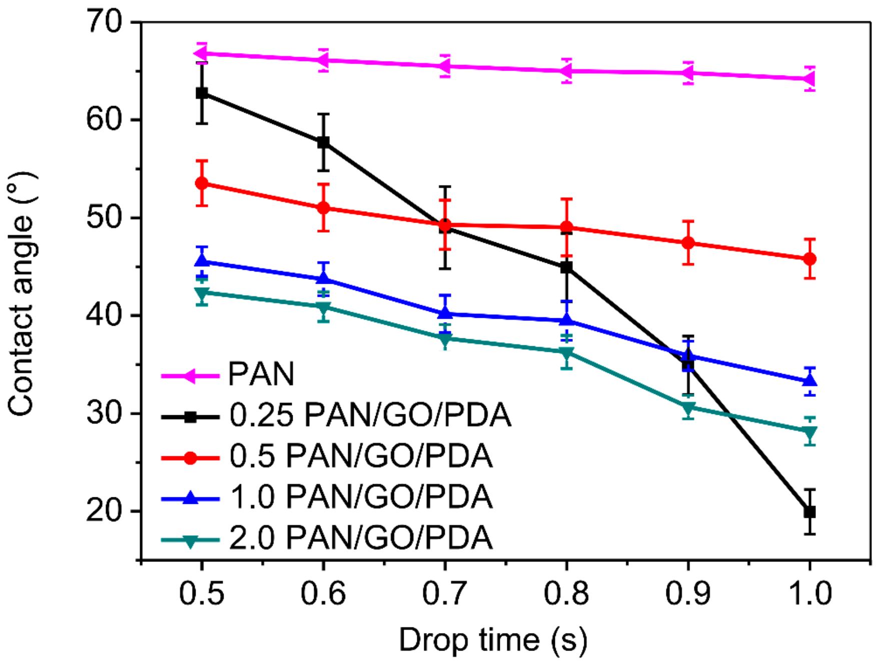

Figure S2. Water contact angles of composite membranes along with drop time, including PAN, 0.25 PAN/GO/PDA, 0.5 PAN/GO/PDA, 1.0 PAN/GO/PDA and 2.0 PAN/GO/PDA membranes.

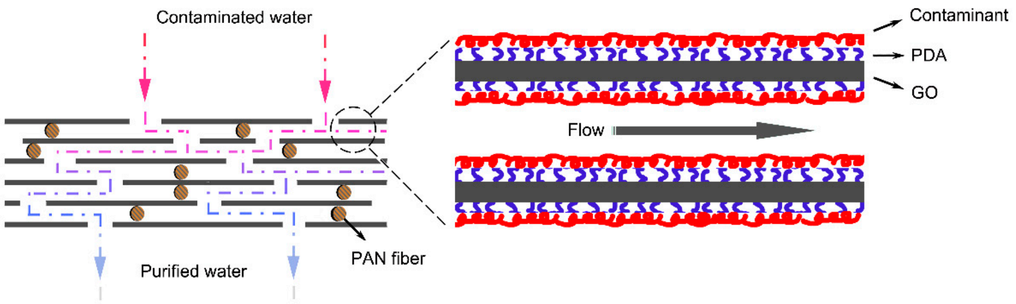

Figure S3. Schematic diagram of gravity driven filtration.

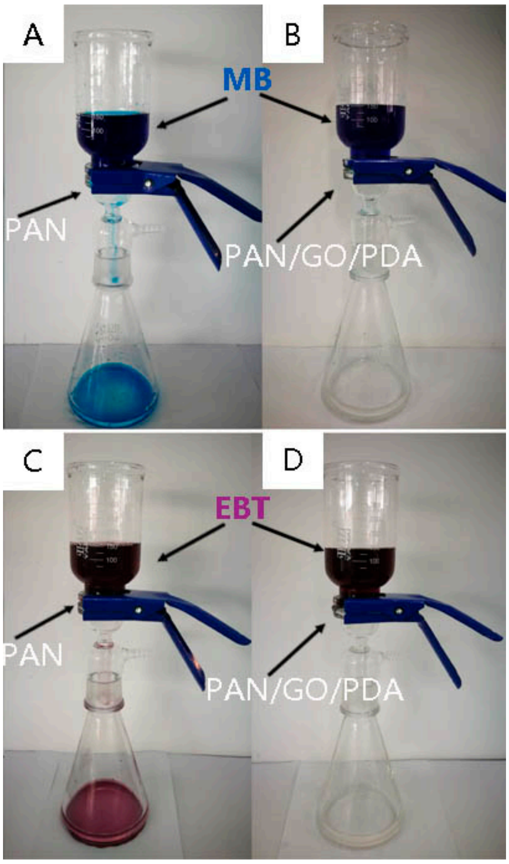

Figure S4. Comparison of filtration effect between ordinary PAN film and PAN/GO/PDA film without external pressure. （A） and （B） show the results of PAN and PAN/GO/PDA adsorbing MB, respectively; （C） and （D） show the results of PAN and PAN/GO/PDA adsorbing EBT, respectively.

For the study of adsorption isotherms, the dye solution (100 mL) (MB pH = 10 or EBT pH = 1) with different concentrations (range from 30 to 200 mg L^{-1}) was mixed with membranes 2.0 PAN/GO/PDA (10 mg). then, the container was transferred into a thermostatic shaker, which was operated at a rotate speed of 150 rpm and a temperature of 25℃ for 12 h. The following equation was used for the calculation of absorbed amount of dye at equilibrium:

$$q_e = \frac{(C_0 - C_e)V}{m} \tag{S1}$$

where C_0 (mg L^{-1}) is the initial concentration of dye; V (L) is the volume of the solution and m (g) is the mass of the membrane; q_e (mg g^{-1}) and C_e (mg L^{-1}) are the equilibrium adsorbed amount and concentration of dye, respectively.

Langmuir model:

$$\frac{C_e}{q_e} = \frac{(1 + C_e K_L)}{q_m K_L} \tag{S2}$$

Freundlich model:

$$\ln q_e = \ln K_F + \frac{1}{n} \ln C_e \tag{S3}$$

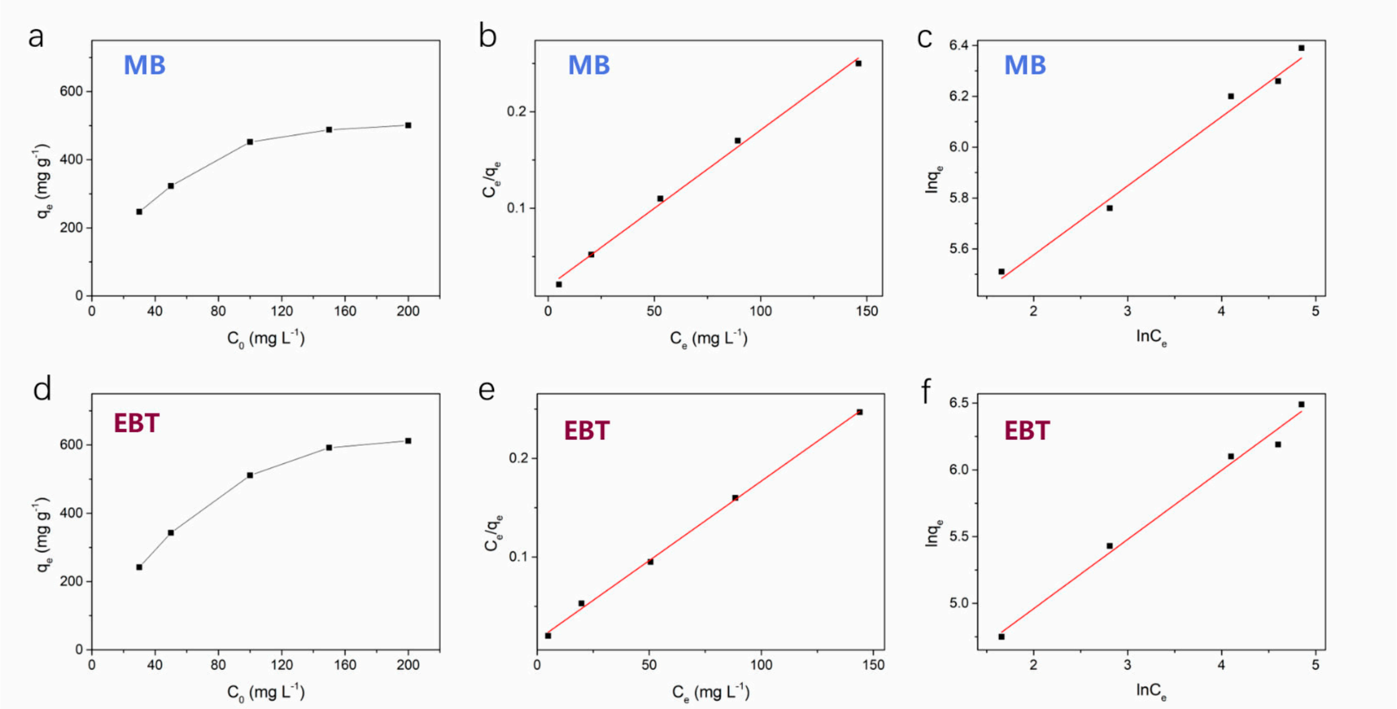

Figure S5. (**a**) and (**d**) represent the variation of equilibrium adsorption capacity versus initial concentration of MB (pH 10) and EBT (pH 1) solution, respectively; (**b**) and (**c**) represent the Langmuir and Freundlich isothermal models for the adsorption of MB, respectively; (**e**) and (**f**) represent the Langmuir and Freundlich isothermal models for the adsorption of EBT, respectively.

As shown as Figure S5, it's clearly seen that either in adsorbing MB or in adsorbing EBT, the Langmuir model exhibits larger R^2, which indicates that the Langmuir model is more suitable in describing the adsorption behavior of organic dyes by the 2.0 PAN/GO/PDA sample. Beyond that, the experiment also shows the maximum adsorption capacity toward MB and EBT, respectively. The results are listed in Table S1 and S2 and compared with other adsorbents.

Table S1. Evaluated model parameters of the adsorption isotherms for MB onto 2.0 PAN/GO/PDA membrane.

Iostherms	Langmiur	Freundlich
Parameters	q_m = 491.4 mg g^{-1}	1/n = 0.25634
	K_L = 0.07178 L mg^{-1}	K_F = 155.8896 L mg^{-1}
	R^2 = 0.9944	R^2 = 0.9883

Table S2. Evaluated model parameters of the adsorption isotherms for EBT onto 2.0 PAN/GO/PDA membrane.

Iostherms	Langmiur	Freundlich
Parameters	q_m = 613.5 mg g^{-1}	1/n = 1.2512
	K_L = 0.0165 L mg^{-1}	K_F = 67.4561 L mg^{-1}
	R^2 = 0.9979	R^2 = 0.9844

Table S3. XPS spectra peak position of C1s, N1s and O1s and atomic ratios of PAN/GO NFMs and PAN/GO/PDA NFMs.

Name	Peak position (eV)	Atomic ratio (PAN/GO)	Atomic ratio (PAN/GO/PDA)
C1s	285.3	74.44	72.41
O1s	532.1	12.80	10.70
N1s	339.5	12.76	16.89

Table S4. Comparison of mechanical properties of PAN/GO-PDA composite membranes and other typically reported electrospinning membranes.

Materials	Tensile strength (MPa)	Young's modulus (MPa)	Refs.
PAN electrospun membrane	~4.6	~75	[1]
PAN NFMs modified with PDA	~10.5	~165	[1]
PSu NFMs modified with PDA	~2.2	~40	[1]
PAN NFMs modified with PVDF particles	7.7	106	[2]
PAN /JCNs composite membrane	~14	-	[3]
PAN-GO-SiO_2 membrane	~7.8	~128	[4]
PEO/PAN/GO	4.08	124	[5]
PNPMs	7.6	-	[6]
PAN/Mt	~14.75	-	[7]
PAN-PVC/MWCNT	2.84	90.70	[8]
TPU/ACNT/AgNPs/PDMS	12.26	4.03	[9]
PD5T5 membrane	11.68	-	[10]
YAG membrane	5.63	-	[11]
PLA/TiO_2 nfs fabrics	3.68	97.32	[12]
PLA/SSS	~4.85	~145.6	[13]
2.0 PAN/GO/PDA composite membrane	~16.8	~232.1	*This work*

Table S5. Comparison of the maximum adsorption capacities (q_m, mg/g) toward MB and EBT between the adsorbent reported in literature and the composite PAN/GO/PDA nanofiber membrane in this work. The maximum adsorption capacities were calculated through the Langmiur adsorption model.

**MB adsorption**			**EBT adsorption**		
Adsorbent	q_m (mg/g)	Ref.	Adsorbent	q_m (mg/g)	Ref.
m-ALG/RH	344	[14]	Magnetite/pectin NPs	103.41	[15]
BPCMC-g-poly (NaAc-co-AM)	333.3	[16]	mLCSCar0.2	280	[17]
Fe_3O_4-xGO	526.32	[18]	Magnetic graphene oxide	210.53	[19]
mGO/PVA-CG	270.94	[20]	CNU2	650.34	[21]
PPy/TiO_2	273.22	[22]	Activated clay	53.95	[23]
PSSC	136.99	[24]	DXC	47.02	[25]
GO/CA	974.7	[26]	MSGT	167.01	[27]
Graphene	153.85	[28]	B-CuFe-CS	806.4	[29]
PVDF/PDA/PPy	370.4	[30]	MgAl	540.91	[31]
2.0 PAN/GO/PDA	491.4	*This work*	2.0 PAN/GO/PDA	613.5	*This work*

Table S6. Comparison of the adsorption capacities toward Cu^{2+} between the adsorbent reported in literature and the composite 2.0 PAN/GO/PDA nanofiber membrane in this work.

Adsorbent	pH	Adsorption capacity (mg g^{-1})	Ref.
PANI/Fe_3O_4-polythersulfone	6.0	104.45	[32]
MWCNT-NH_2-CS/PVA	5.5	20.1	[33]
PAN/Fe_3O_4@Fe_3O_4	6.0	35 ± 2	[34]
MWCNT-PEI/PAN	7.0	112.5	[35]
aPAN/BPEI NMs	6	209.53	[36]
GO/PPy/MnO_2	-	38.4	[37]
PVA/ GO nanofiber film	7	57.88	[38]
PGCB	4	83.3	[39]
CS-CD	-	49.97	[40]
2.0 PAN/GO/PDA	4.78	62.9	*This work*

Porosity calculation equation:

$$porosity(\varepsilon)\% = \frac{(m_w - m_d)/\rho_w}{\frac{m_w - m_d}{\rho_w} + \frac{m_d}{\rho}} \times 100\% \quad \text{(S4)}$$

Where m_w and m_d are the mass of wet and dry membrane respectively, ρ_w and ρ are density of water and PAN.

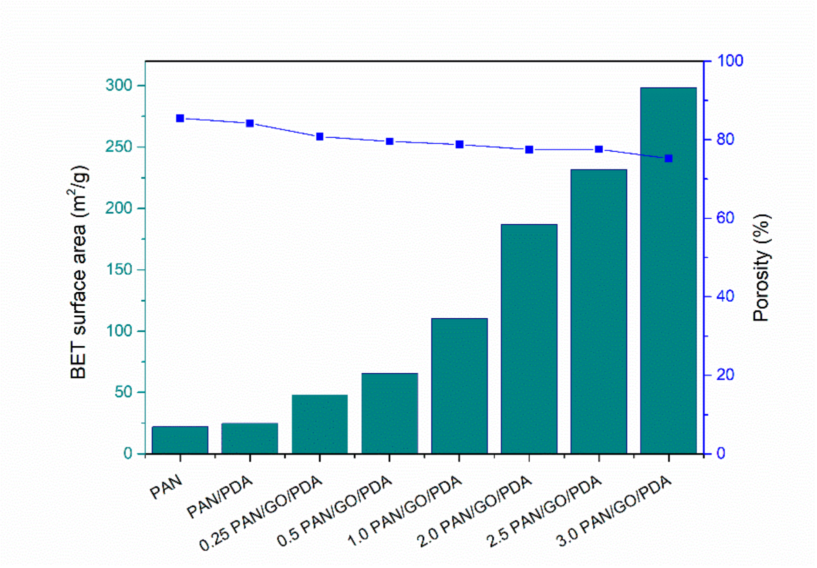

Figure S6. BET surface area and porosity results of different membranes.

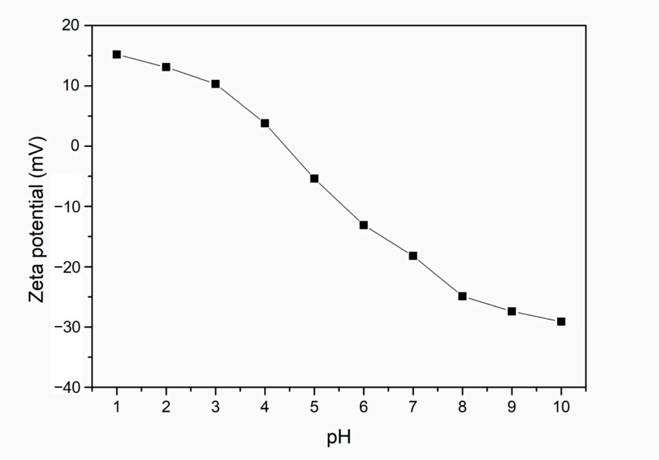

Figure S7. Zeta-potential of sample 2.0 PAN/GO/PDA under different pH values.

References

1. Huang, L.; Arena, J.T.; Manickam, S.S.; Jiang, X.; Willis, B.G.; McCutcheon, J.R. Improved mechanical properties and hydrophilicity of electrospun nanofiber membranes for filtration applications by dopamine modification. *J. Membr. Sci.* **2014**, *460*, 241–249, https://doi.org/10.1016/j.memsci.2014.01.045.
2. Elkhaldi, R.M.; Guclu, S.; Koyuncu, I. Enhancement of mechanical and physical properties of electrospun PAN nanofiber membranes using PVDF particles. *DESALINATION Water Treat.* **2016**, *57*, 26003–26013, https://doi.org/10.1080/19443994.2016.1159253.
3. Cao, X.; Huang, M.; Ding, B.; Yu, J.; Sun, G. Robust polyacrylonitrile nanofibrous membrane reinforced with jute cellulose nanowhiskers for water purification. *Desalination* **2013**, *316*, 120–126, https://doi.org/10.1016/j.desal.2013.01.031.
4. N. Naseeb, A.A. Mohammed, T. Laoui, Z. Khan, A Novel PAN-GO-SiO2 Hybrid Membrane for Separating Oil and Water from Emulsified Mixture, *Materials*, **2019**, 12, 212, 10.3390/ma12020212
5. Abdollahi, S.; Ehsani, M.; Morshedian, J.; Khonakdar, H.A.; Reuter, U. Structural and electrochemical properties of PEO/PAN nanofibrous blends: Prediction of graphene localization. *Polym. Compos.* **2017**, *39*, 3626–3635, https://doi.org/10.1002/pc.24390.
6. Dong, X.; Zheng, Y.; Xin, B.; Lin, L.; Zhang, F. Preparation and characterization of composite fibrous membranes for oil spill cleanup. *Text. Res. J.* **2019**, *90*, 313–322, https://doi.org/10.1177/0040517519865042.
7. Bansal, P.; Batra, R.; Yadav, R.; Purwar, R. Electrospun polyacrylonitrile nanofibrous membranes supported with montmorillonite for efficient PM 2.5 filtration and adsorption of Cu (II) ions. *J. Appl. Polym. Sci.* **2021**, *139*, https://doi.org/10.1002/app.51582.
8. J. Namsaeng, W. Punyodom, P. Worajittiphon, Synergistic effect of welding electrospun fibers and MWCNT re-inforcement on strength enhancement of PAN–PVC non-woven mats for water filtration, *Chemical Engineering Science*, **2019**, 193, 230-242, https://doi.org/10.1016/j.ces.2018.09.019
9. L. Lin, L. Wang, B. Li, J. Luo, X. Huang, Q. Gao, H. Xue, J. Gao, Dual conductive network enabled superhydro-phobic and high performance strain sensors with outstanding electro-thermal performance and extremely high gauge factors, *Chemical Engineering Journal*, **2020**, 385,123391, https://doi.org/10.1016/j.cej.2019.123391
10. Chee, T.Y.; Yusoff, A.R.M.; Abdullah, F.; Mahmood, W.M.A.W.; Jasni, M.J.F.; Malek, N.A.N.N.; Buang, N.A.; Govarthanan, M. Fabrication, characterization and application of electrospun polysulfone membrane for phosphate ion removal in real samples. *Chemosphere* **2022**, *303*, https://doi.org/10.1016/j.chemosphere.2022.135228.
11. J. Jiang, N. Ni, W. Xiao, X. Zhao, F. Guo, X. Fan, Q. Ding, W. Hao, P. Xiao, Robust ceramic nanofibrous mem-branes with ultra-high water flux and nanoparticle rejection for self-standing ultrafiltration, *Journal of the European Ceramic Society*, **2021**, 41, 4264-4272, https://doi.org/10.1016/j.jeurceramsoc.2021.02.012
12. Yu, Q.; Qin, Y.; Han, M.; Pan, F.; Han, L.; Yin, X.; Chen, Z.; Wang, L.; Wang, H. Preparation and characterization of solvent-free fluids reinforced and plasticized polylactic acid fibrous membrane. *Int. J. Biol. Macromol.* **2020**, *161*, 122–131, https://doi.org/10.1016/j.ijbiomac.2020.06.027.
13. C.S. Wu, D.Y. Wu, S.S. Wang, Bio-based polymer nanofiber with siliceous sponge spicules prepared by electro-spinning: Preparation, characterisation, and functionalisation, *Materials Science and Engineering: C*, **2020**, 108, 110506, https://doi.org/10.1016/j.msec.2019.110506.
14. Alver, E.; Metin, A..; Brouers, F. Methylene blue adsorption on magnetic alginate/rice husk bio-composite. *Int. J. Biol. Macromol.* **2020**, *154*, 104–113, https://doi.org/10.1016/j.ijbiomac.2020.02.330.
15. Attallah, O.A.; Al-Ghobashy, M.A.; Nebsen, M.; Salem, M.Y. Removal of cationic and anionic dyes from aqueous solution with magnetite/pectin and magnetite/silica/pectin hybrid nanocomposites: kinetic, isotherm and mechanism analysis. *RSC Adv.* **2016**, *6*, 11461–11480, https://doi.org/10.1039/c5ra23452b.

16. Bello, K.; Sarojini, B.K.; Narayana, B.; Rao, A.; Byrappa, K. A study on adsorption behavior of newly synthesized banana pseudo-stem derived superabsorbent hydrogels for cationic and anionic dye removal from effluents. *Carbohydr. Polym.* **2018**, *181*, 605–615, https://doi.org/10.1016/j.carbpol.2017.11.106.
17. M.H. Karimi, G.R. Mahdavinia, B. Massoumi, A. Baghban, M. Saraei, Ionically crosslinked magnetic chi-tosan/kappa-carrageenan bioadsorbents for removal of anionic eriochrome black-T, International Journal of Biolog-ical *Macromolecules*, **2018**, 113, 361-375, https://doi.org/10.1016/j.ijbiomac.2018.02.102.
18. Cui, L.; Guo, X.; Wei, Q.; Wang, Y.; Gao, L.; Yan, L.; Yan, T.; Du, B. Removal of mercury and methylene blue from aqueous solution by xanthate functionalized magnetic graphene oxide: Sorption kinetic and uptake mechanism. *J. Colloid Interface Sci.* **2015**, *439*, 112–120, https://doi.org/10.1016/j.jcis.2014.10.019.
19. Khurana, I.; Shaw, A.K.; Bharti; Khurana, J.M.; Rai, P.K. Batch and dynamic adsorption of Eriochrome Black T from water on magnetic graphene oxide: Experimental and theoretical studies. *J. Environ. Chem. Eng.* **2018**, *6*, 468–477, https://doi.org/10.1016/j.jece.2017.12.029.
20. Z. Cheng, J. Liao, B. He, F. Zhang, F. Zhang, X. Huang, L. Zhou, One-Step Fabrication of Graphene Oxide En-hanced Magnetic Composite Gel for Highly Efficient Dye Adsorption and Catalysis, *ACS Sustainable Chemistry & Engineering*, **2015**, 3, 1677-1685, https://doi.org/10.1021/acssuschemeng.5b00383.
21. Nguyen, P.; Ho, K.; Do, N.; Nguyen, C.; Nguyen, H.; Tran, K.; Le, K.; Le, P. A comparative study on modification of aerogel-based biosorbents from coconut fibers for treatment of dye- and oil-contaminated water. *Mater. Today Sustain.* **2022**, *19*, https://doi.org/10.1016/j.mtsust.2022.100175.
22. Li, J.; Feng, J.; Yan, W. Excellent adsorption and desorption characteristics of polypyrrole/TiO2 composite for Methylene Blue. *Appl. Surf. Sci.* **2013**, *279*, 400–408, https://doi.org/10.1016/j.apsusc.2013.04.127.
23. Nkwoada, A.; Onyedika, G.; Oguzie, E.; Ogwuegbu, M. Thermodynamics, Kinetics, and Reaction Mechanism of Kaolin Adsorption/Photocatalysis of Hazardous Cationic and Anionic Dyes. *Chem. Afr.* **2022**, 1–16, https://doi.org/10.1007/s42250-022-00426-0.
24. X. Li, H. Lu, Y. Zhang, F. He, Efficient removal of organic pollutants from aqueous media using newly synthe-sized polypyrrole/CNTs-CoFe2O4 magnetic nanocomposites, Chemical Engineering Journal, 2017, 316, 893-902, https://doi.org/10.1016/j.cej.2017.02.037.
25. Sriram, G.; Uthappa, U.; Rego, R.M.; Kigga, M.; Kumeria, T.; Jung, H.-Y.; Kurkuri, M.D. Ceria decorated porous diatom-xerogel as an effective adsorbent for the efficient removal of Eriochrome Black T. *Chemosphere* **2019**, *238*, 124692, https://doi.org/10.1016/j.chemosphere.2019.124692.
26. F. Zhao, Y. Zhang, X. Zhang, L. Zhao, F. Fu, B. Mu, A. Wang, Preparation of efficient adsorbent with dual ad-sorption function based on semi-coke: Adsorption properties and mechanisms, *Journal of Colloid and Interface Science*, **2022**, 626, 674-686, DOI: 10.1016/j.jcis.2022.06.100.
27. V.M. Vučurović, R.N. Razmovski, U.D. Miljić, V.S. Puškaš, Removal of cationic and anionic azo dyes from aqueous solutions by adsorption on maize stem tissue, *Journal of the Taiwan Institute of Chemical Engineers*, **2014**, 45, 1700-1708, https://doi.org/10.1016/j.jtice.2013.12.020.
28. Liu, T.; Li, Y.; Du, Q.; Sun, J.; Jiao, Y.; Yang, G.; Wang, Z.; Xia, Y.; Zhang, W.; Wang, K.; et al. Adsorption of methylene blue from aqueous solution by graphene. *Colloids Surfaces B: Biointerfaces* **2012**, *90*, 197–203, https://doi.org/10.1016/j.colsurfb.2011.10.019.
29. M. Zubair, H.A. Aziz, I. Ihsanullah, M.A. Ahmad, M.A. Al-Harthi, Enhanced removal of Eriochrome Black T from water using biochar/layered double hydroxide/chitosan hybrid composite: Performance evaluation and opti-mization using BBD-RSM approach, *Environmental Research*, **2022**, 209, 112861, https://doi.org/10.1016/j.envres.2022.112861.
30. F.-f. Ma, D. Zhang, N. Zhang, T. Huang, Y. Wang, Polydopamine-assisted deposition of polypyrrole on electro-spun poly(vinylidene fluoride) nanofibers for bidirectional removal of cation and anion dyes, *Chemical Engineering Journal*, **2018**, 354, 432-444, https://doi.org/10.1016/j.cej.2018.08.048.
31. M. Zubair, N. Jarrah, M.S. Manzar, M. Al-Harthi, M. Daud, N.D. Mu'azu, S.A. Haladu, Adsorption of erio-chrome black T from aqueous phase on MgAl-, CoAl- and NiFe- calcined layered double hydroxides: Kinetic, equi-librium and thermodynamic studies, *Journal of molecular liquids*, **2017**, 230, 344-352, https://doi.org/10.1016/j.molliq.2017.01.031.
32. Daraei, P.; Madaeni, S.S.; Ghaemi, N.; Salehi, E.; Khadivi, M.A.; Moradian, R.; Astinchap, B. Novel polyethersulfone nanocomposite membrane prepared by PANI/Fe3O4 nanoparticles with enhanced performance for Cu(II) removal from water. *J. Membr. Sci.* **2012**, *415-416*, 250–259, https://doi.org/10.1016/j.memsci.2012.05.007.
33. Salehi, E.; Madaeni, S.; Rajabi, L.; Vatanpour, V.; Derakhshan, A.; Zinadini, S.; Ghorabi, S.; Monfared, H.A. Novel chitosan/poly(vinyl) alcohol thin adsorptive membranes modified with amino functionalized multi-walled carbon nanotubes for Cu(II) removal from water: Preparation, characterization, adsorption kinetics and thermodynamics. *Sep. Purif. Technol.* **2012**, *89*, 309–319, https://doi.org/10.1016/j.seppur.2012.02.002.
34. K.E. Greenstein, N.V. Myung, G.F. Parkin, D.M. Cwiertny, Performance comparison of hematite (al-pha-Fe2O3)-polymer composite and core-shell nanofibers as point-of-use filtration platforms for metal sequestration, *Water Res*, **2019**, 148, 492-503, DOI: 10.1016/j.watres.2018.10.048.
35. Deng, S.; Liu, X.; Liao, J.; Lin, H.; Liu, F. PEI modified multiwalled carbon nanotube as a novel additive in PAN nanofiber membrane for enhanced removal of heavy metal ions. *Chem. Eng. J.* **2019**, *375*, https://doi.org/10.1016/j.cej.2019.122086.

36. H. Shao, D. Yin, D. Li, Q. Ma, W. Yu, X. Dong, Simultaneous visual detection and removal of Cu(2+) with elec-trospun self-supporting flexible amidated polyacrylonitrile/branched polyethyleneimine nanofiber membranes, *ACS Applied Materials & Interfaces*, **2021**, 13, 49288-49300, https://doi.org/10.1021/acsami.1c13722.
37. Y. Zhang, Y. Wang, J. Xue, C. Tang, MnO2-coated graphene/polypyrrole hybrids for enhanced capacitive deion-ization performance of Cu2+ removal, *Industrial & Engineering Chemistry Research*, **2022**, 61, 3582-3590, https://doi.org/10.1021/acs.iecr.1c04159.
38. Y. He, H. Tian, A. Xiang, S. Ma, D. Yin, A. Varada Rajulu, Fabrication of PVA/GO Nanofiber films by electro-spinning: Application for the adsorption of Cu2+ and organic dyes, *Journal of Polymers and the Environment*, **2022**, 30, 2964-2975, DOI:10.1007/s10924-021-02366-1.
39. Igberase, E.; Osifo, P.; Ofomaja, A. The adsorption of copper (II) ions by polyaniline graft chitosan beads from aqueous solution: Equilibrium, kinetic and desorption studies. *J. Environ. Chem. Eng.* **2014**, *2*, 362–369, https://doi.org/10.1016/j.jece.2014.01.008.
40. Pan, L.; Wang, C.; Wu, W.; Li, X.; Ma, S.; Li, C.; Shen, Y.; Ou, J. Bioinspired honeycomb-like 3D architectures self-assembled from chitosan as dual-functional membrane for effective adsorption and detection of copper ion. *Microporous Mesoporous Mater.* **2022**, *335*, 111859, https://doi.org/10.1016/j.micromeso.2022.111859.
